# A modified Delphi to inform development of a multidimensional diet quality score for a sustainable healthy diet

**DOI:** 10.1017/S0007114526106345

**Published:** 2026-05-28

**Authors:** Emily Denniss, Mark Lawrence, Sarah A. McNaughton, Katherine Livingstone, Priscila Machado

**Affiliations:** 1 School of Health and Social Development, Institute for Physical Activity and Nutrition, Deakin University, Geelong, Australia; 2 School of Exercise and Nutrition Sciences, Institute for Physical Activity and Nutrition, Deakin University, Geelong, Australia; 3 School of Human Movement and Nutrition Sciences, Health and Well-Being Centre for Research Innovation, The University of Queensland, St Lucia, Australia; 4 School of Exercise and Nutrition Sciences, Deakin University, Geelong, Australia

**Keywords:** Diet quality metrics, sustainable healthy diets, sustainability, dietary variety, animal products, ultra-processed foods

## Abstract

No existing dietary metric simultaneously captures key dimensions of sustainable healthy diets (SHD): dietary variety, intake of animal products and extent of food processing. This methods and construct development study aimed to identify indicators of an SHD that can be used to inform a multidimensional diet quality score. A modified Delphi was used to gain expert consensus regarding the development of an SHD score. Three iterative surveys were conducted between November 2022 and May 2023. Surveys asked participants’ opinions regarding the measurement of the three dimensions of SHD (Dimension 1: variety of unprocessed and minimally processed foods; Dimension 2: intake of animal products; and Dimension 3: intake of ultra-processed foods (UPF)) and weighting and aggregation of a score that assesses these three dimensions. Thirteen international experts completed all three surveys. Consensus from experts led to the identification of food-based indicators of SHD. Experts agreed that Dimension 1 should be comprised of twelve food groups, with food groups and scoring ranges informed by the Global Diet Quality Score; Dimension 2 comprised of five food groups with scoring ranges informed by the EAT-Lancet planetary health diet; and Dimension 3 as one food group measured as a cut-off value of ≤10 % energy from UPF. There was consensus that each dimension should be equally weighted. Outcomes from this work have been used to inform the development and validation of a multidimensional diet quality score to assess the healthfulness and environmental sustainability of diets among healthy adult populations.

Diet-related non-communicable diseases and environmental degradation are urgent societal challenges of the twenty-first century^(1)^. These global challenges share unhealthy and unsustainable diets as underlying drivers^(2,3)^. Promoting sustainable healthy diets (SHD) is essential to achieving the Sustainable Development Goals of the UN^(1)^. Acknowledging this high priority and the existence of diverging views on the concepts of SHD, the FAO and the WHO launched a report on guiding principles to define SHD. These are defined as ‘*dietary patterns that promote all dimensions of health and wellbeing; have low environmental pressure and impact; are accessible, affordable, safe and equitable; and are culturally acceptable*’^(4)^.

The FAO/WHO report highlights three key dimensions of SHD. First, SHD are based on a great variety of unprocessed and minimally processed foods; second, they include moderate amounts of eggs, dairy products, poultry, fish and small amounts of red meat; and third, they restrict consumption of highly processed foods (or ultra-processed foods (UPF))^(4,5)^. In a scoping review conducted by the research team, forty-eight food-based, investigator-defined metrics assessing diet quality in free-living, healthy populations at the individual or household level were assessed^(6)^. A range of metrics has been developed to measure the healthiness of diets (e.g. Diet Quality Index), yet no existing metric simultaneously assesses the three dimensions of SHD recommended by FAO/WHO^(6,7)^. Emerging evidence has shown that some plant-based diets may be more sustainable and decrease chronic disease risk^(8–11)^. However, evidence is conflicting, which may be due to the inclusion of unhealthy foods, such as UPF (e.g. soft drinks, industrial frozen meals and ‘alternative’ meats) as part of these diets, and/or lack of consideration of dietary variety^(12,13)^.

Recently, several metrics have been designed to capture aspects of SHD, such as the Sustainable Diet Index (SDI)^(14)^ and the Healthy and Sustainable Diet Index (HSDI)^(15)^. However, these metrics have limitations. For example, existing metrics require a set of instruments for measuring different aspects of the environmental, nutritional, economic and sociocultural aspects of diets. The SDI requires imputing data from several sources, with a diet quality index (PANDiet) being one of the indicators^(14)^, whereas the HSDI includes edible plate waste and single-use package^(15)^. These additional indicators are not captured in commonly used dietary assessment methods such as 24-h recalls and FFQ. A further limitation of the HSDI is that food intakes are measured using the Australian Dietary Guidelines as the reference, which does not align with all SHD principles^(6,15)^. Despite its relevance, the EAT-Lancet planetary health diet paid limited attention to the degree of processing^(6)^. Fardet and Rock proposed the 3V-based diet (*Végétal* (plant), *Vrai* (real) and *Varié* (varied)), drawing from scientific literature and modelling based on French serving sizes^(16)^. The 3V-based diet recommends ≤15 % of daily energy intake from animal foods and ≤15 % UPF; however, it does not propose an overall diet quality score or food group cut-offs to measure dietary variety or intake of animal products based on global recommendations^(16)^.

The absence of appropriate metrics for SHD limits the assessment of the healthfulness and environmental impact of dietary patterns, which limits the body of evidence that would otherwise inform national and international policy actions^(6)^. In the absence of a gold standard to measure global SHD, novel dietary metrics could be developed considering the key dimensions for SHD when using food group consumption as representations of constructs of diet quality: dietary variety, intake of animal products and extent of food processing^(6)^. Drawing on the premise that these are the three key dimensions of SHD, this methods and construct development study aimed to identify indicators for each dimension of an SHD that can be used to inform a multidimensional diet quality score intended for global application. Key outcomes of this research include a transparent expert-informed set of dietary indicators for the three dimensions of SHD and agreed upon scoring and weighting procedures to underpin a diet quality score for measuring SHD.

## Method

### Study design

This study used a modified Delphi method, which involved iterative surveys of experts and followed the Conducting and REporting of DElphi Studies (CREDES) framework (Supplementary Table 1)^(17)^. The Delphi method is often used in public health and nutrition to gain expert consensus^(18–20)^ and is appropriate to use when there is incomplete knowledge or contested views on a topic^(21)^. It has been successfully used to develop tools, guidelines and definitions for use in research and practice^(19,22,23)^. Delphi studies are useful for managing panel deliberations and ensure that equal weight is given to the opinion of each respondent, while maintaining anonymity^(24)^. As recommended in the Delphi literature, the number of survey iterations was decided at the beginning of the study^(21)^. Three surveys are considered optimal, and therefore, the number of iterations was set at 3^(21)^.

The surveys were conceptually guided by the FAO/WHO report on guiding principles of SHD^(4)^, the NOVA system^(25,26)^, a food classification that categorises foods according to the extent and purpose of industrial food processing, and recommendations on the appropriate construction features for dietary indices by Burggraf et al.^(27)^. There is agreement among experts about the dimensions of SHD^(4)^, as shown in Table 1. An important premise was that dietary variety, intake of animal products and extent of food processing are key dimensions of SHD, and thus experts were asked to provide feedback about how to operationalise these dimensions using food group indicators to inform the development of a new diet quality score. The definitions (Table 1), metrics and scoring procedures presented in the Delphi surveys were informed by a scoping review of metrics for assessing SHD^(6)^. It is best practice for a literature review to inform the first survey of a modified Delphi^(20)^.

**Table 1.** Definitions and dimensions of sustainable healthy diets informing the Delphi study Table 1 long description.

Definitions	
Sustainable healthy diets	Dietary patterns that promote all dimensions of health and wellbeing; have low environmental pressure and impact; are accessible, affordable, safe and equitable; and are culturally acceptable^(4)^.
Dietary patterns	Consider the combinations, types and amounts of foods consumed in the diet on a regular basis^(42)^.
Dietary metric/diet quality score	Measures of investigator-defined food-based dietary patterns (also referred to as ‘*a priori*’ methods). Reflect previous knowledge, guidelines and recommendations using pre-specified criteria, for example, adherence to health and/or environmental sustainability-based recommendations^(43)^.
Unprocessed and minimally processed foods	Defined within the NOVA system as edible parts of plants, animals, fungi, algae and water, and natural foods altered by processes designed to preserve natural foods, to make them suitable for storage, or to make them safe, edible or more pleasant to consume without adding food substances to the original food^(26)^.
Ultra-processed foods	Defined within the NOVA system as formulations of ingredients, mostly of exclusive industrial use, that result from a series of industrial processes (hence ‘ultra-processed’)^(26)^. These products contain little if any whole food; examples include soft drinks, fast-food dishes, confectionery and industrial frozen meals.
**Dimensions of sustainable healthy diets**	
Dimension 1: Sustainable healthy diets are based on a great variety of unprocessed or minimally processed foods^(4)^.	
Dimension 2: Sustainable healthy diets can include moderate amounts of eggs, dairy products, poultry and fish and small amounts of red meat^(4)^.	
Dimension 3: Sustainable healthy diets restrict consumption of ultra-processed foods^(4)^.	

### Participants and recruitment

International experts in nutritional epidemiology, environmental health, dietary assessment and/or food and nutrition policy were invited to participate. Three recruitment strategies were used. First, the research team developed a list of individuals who were known through their professional networks as having expertise in one or more of the relevant fields. Second, literature searches in PubMed for studies related to SHD and dietary assessment methods were used to identify first and senior authors of relevant articles. Identified authors were searched in Google to determine if they had a background and training in nutrition or environmental sustainability. Third, snowballing was used and individuals who agreed to participate were asked to suggest others who may have relevant expertise. Recent publications by the identified individuals were also checked to determine if they had conflicts of interest that may impact their responses (e.g. food industry funding). Individuals with publicly available email addresses and no identified conflicts of interest were sent an email invitation and link to the first survey. In total, seventy-eight experts were invited, fifty-eight identified through literature searches, nineteen identified by the research team and one through snowballing. In the Delphi literature, a minimum sample size of 8 has been recommended when participants have specific knowledge of the topic^(21)^.

### Data collection

**Figure 1.**
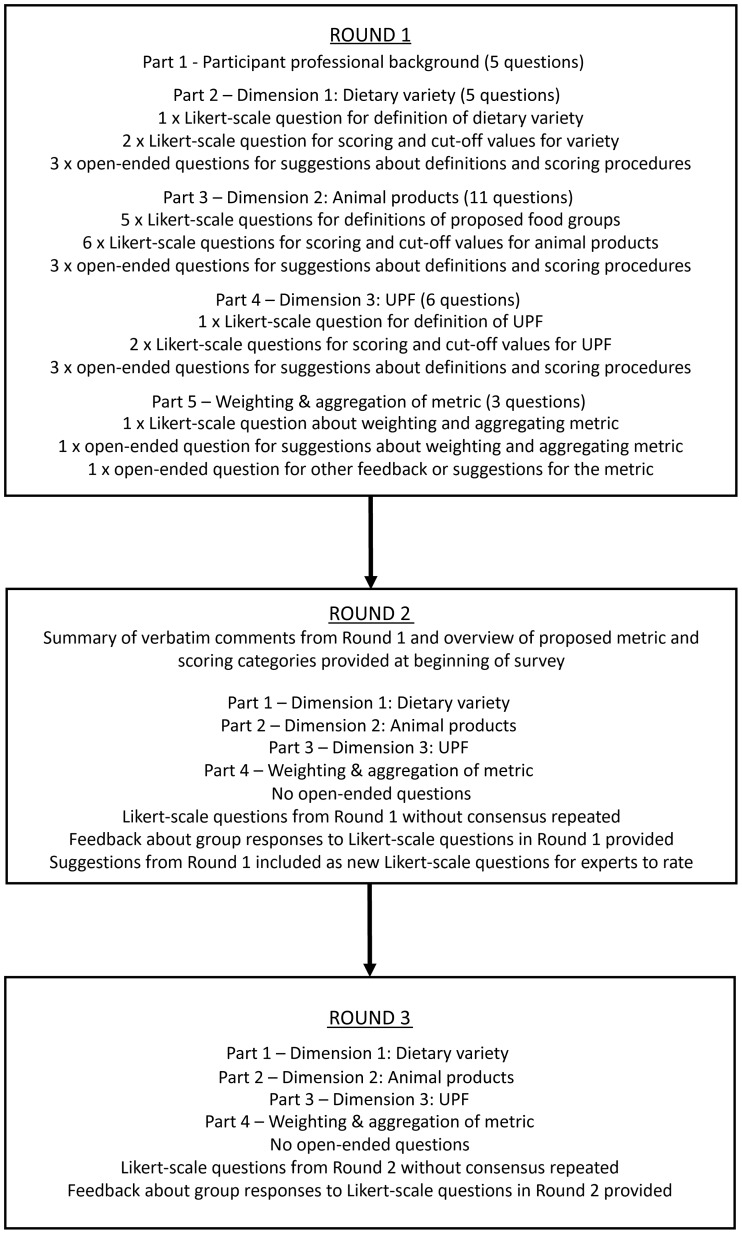
Modified Delphi method and structure of each survey round. UPF, ultra-processed food.

Participants were invited to complete three iterative surveys, herein referred to as rounds, administered via Qualtrics. Surveys were conducted between November 2022 and May 2023. The modified Delphi method and structure of each round are summarised in Figure 1. Participants who consented but did not complete a round were considered to have dropped out and were not asked to complete the subsequent round (e.g. if Round 1 was not completed, the participant was not asked to complete Round 2).

### Round 1

Round 1 was structured in five parts, including general questions about participants’ professional background, years of experience, education and expertise; one section about each of the three dimensions of SHD; and the final section asked about weighting and aggregating the diet quality score. Round 1 comprised of Likert-scale and open-ended questions. For each component, participants were asked to rate their agreement with proposed definitions and scoring procedures or proposed weighting methods on a five-point Likert scale ranging from strongly agree (5) to strongly disagree (1). Likert-scale questions are advised in the Delphi literature, and scales between 4 and 7 are considered ideal^(21,24)^. Open-ended questions were also included so that participants could provide specific suggestions about the proposed definitions, scoring procedures and weighting procedures. Including open-ended questions in the first survey is a recommended approach in modified Delphi studies^(21)^. Participants were provided with instructions explaining that the purpose was to identify dietary indicators for the free-living adult population, rather than other population groups such as children or pregnant people.

#### Dimension 1: variety of unprocessed and minimally processed foods

Dimension 1 was based on Principle 2 from the FAO/WHO report: *‘SHD are based on a great variety of unprocessed or minimally processed foods, balanced across food groups, while restricting highly processed food and drink products’* ^(4)^. In this section, participants were asked about the variety of unprocessed or minimally processed food components of Principle 2, defined by the NOVA system^(25)^. The proposed food groups were based on the FAO Minimum Dietary Diversity (MDD) indicator, which groups foods that are nutritionally similar and/or play the same role in the diet^(28)^. UPF were not included in the proposed food groups. This dimension included three Likert-scale questions covering the definition of dietary variety (one question), scoring procedures and cut-off values for scoring dietary variety (two questions) and open-ended questions (three questions) (Figure 1).

#### Dimension 2: intake of animal products

Dimension 2 was based on Principle 4 *‘SHD can include moderate amounts of eggs, dairy, poultry and fish; and small amounts of red meat’* ^(4)^. The proposed food groups and thresholds for consumption are consistent with both health and environmental sustainability recommendations and were based on the EAT-Lancet planetary health diet^(29)^ and NOVA system^(25)^. UPF were not included in the proposed food groups. These foods were captured elsewhere as part of Principle 2 to avoid overlapping measures of FAO/WHO principles. Plant-based alternatives (e.g. soy beverages, margarine and plant-based meat) and cellular or cultivated meat, seafood, dairy products and cell-based eggs were also not included in the proposed food groups as the FAO/WHO recommendations are based on current evidence of health and environmental impacts of animal foods. This dimension included eleven Likert-scale questions covering the definitions of egg, dairy products, poultry, fish and red meat (five questions), scoring procedures and cut-off values for intakes of animal products (six questions) and open-ended questions (three questions).

#### Dimension 3: intake of ultra-processed foods

Dimension 3 was based on Principle 2 of the FAO/WHO report^(4)^. Highly processed food and drink products were defined using the NOVA system’s definition of UPF^(25)^. Participants were asked specific questions about restricting the intake of UPF component of Principle 2. This dimension included three Likert-scale questions covering the definition of intake of UPF, scoring procedures and cut-off values for scoring UPF intake and three open-ended questions.

#### Weighting and aggregation of the overall diet quality score

In the final section, participants were asked about weighting and aggregation of the three dimensions of the diet quality score as the number of indicators per dimension differs (e.g. ten groups in Dimension 1, five in Dimension 2 and one in Dimension 3). To aggregate these dimensions into one composite score, some weighting procedures could be considered. Previous research suggests that consideration be given to weighting and aggregation of diet quality scores^(27)^. If equal weighting is given to each food group, this will result in an unequal weighting of the dimensions^(27)^. This section included one Likert-scale question about weighting aggregation and an open-ended question.

### Round 2

After participants completed Round 1, the results were analysed to determine participants’ level of agreement. Responses to open-ended questions were content analysed^(30)^. Similar comments were grouped, and comments containing suggestions regarding the proposed elements of the diet quality score were carried forward into Round 2, so the group could rate their agreement. A summary of all participant comments received in Round 1 was provided verbatim at the beginning of Round 2 for participants to consider. The definitions and scoring procedures were revised based on participant feedback in line with Delphi processes.

A draft of the diet quality score, food groups and scoring categories was presented at the start of Round 2. Participants were presented with the same Likert-scale questions as Round 1, but open-ended questions were omitted. Definitions that participants considered unclear were reworded for clarity, and changes were highlighted. Suggestions about changes to the proposed definitions and scoring procedures were also incorporated into new Likert-scale questions, and participants were asked to rate their agreement. Each repeated Likert-scale question included a bar chart, median response and interquartile range of the participant responses from Round 1. This feedback method for Likert-scale questions is consistently recommended in the literature^(21,24)^. In line with best practice Delphi methods^(30)^, participants were asked to consider how other participants responded before responding again.

### Round 3

Questions from Round 2 that achieved consensus for inclusion in the score were adapted where necessary and excluded from Round 3, and respective contradictory items were also excluded (e.g. if a scoring procedure, such as cut-off, achieved consensus in Round 2, other options of scoring procedures were not presented in Round 3). Otherwise, questions that did not achieve consensus in Round 2 were repeated in Round 3. Questions that did not achieve consensus in Round 3 were deemed not appropriate to be included in the score.

### Consensus

Consensus was set *a priori* at ≥70 % of participants selecting 1 or 2 on the scale (strongly disagree or disagree) or 4 or 5 (agree or strongly agree) and was calculated after Rounds 2 and 3 for each item. While determining consensus varies in Delphi methods, the 70 % cut point was chosen because it has been suggested as an appropriate figure in the Delphi literature^(30)^, and similar cut points (67 %) have been used in public health nutrition Delphi studies^(20,23,31,32)^. Higher cut points for consensus are often used in Delphi studies (e.g. 80 %^(23,33)^). However, a lower cut point of 70 % was selected because participants from a wide range of geographical locations and areas of expertise were invited to participate, and it was anticipated that this may lead to a greater divergence in opinions, compared with Delphi studies that have more homogenous expert panels. Dietary indicators and scoring procedures that achieved consensus were considered appropriate to inform a diet quality score.

### Ethics approval and consent to participate

This study was conducted according to the guidelines laid down in the Declaration of Helsinki, and all procedures involving human participants were approved by the Deakin University Human Ethics Advisory Group (HEAG-H 165_2022). Written informed consent was obtained from all participants.

## Results

### Overview

**Figure 2.**
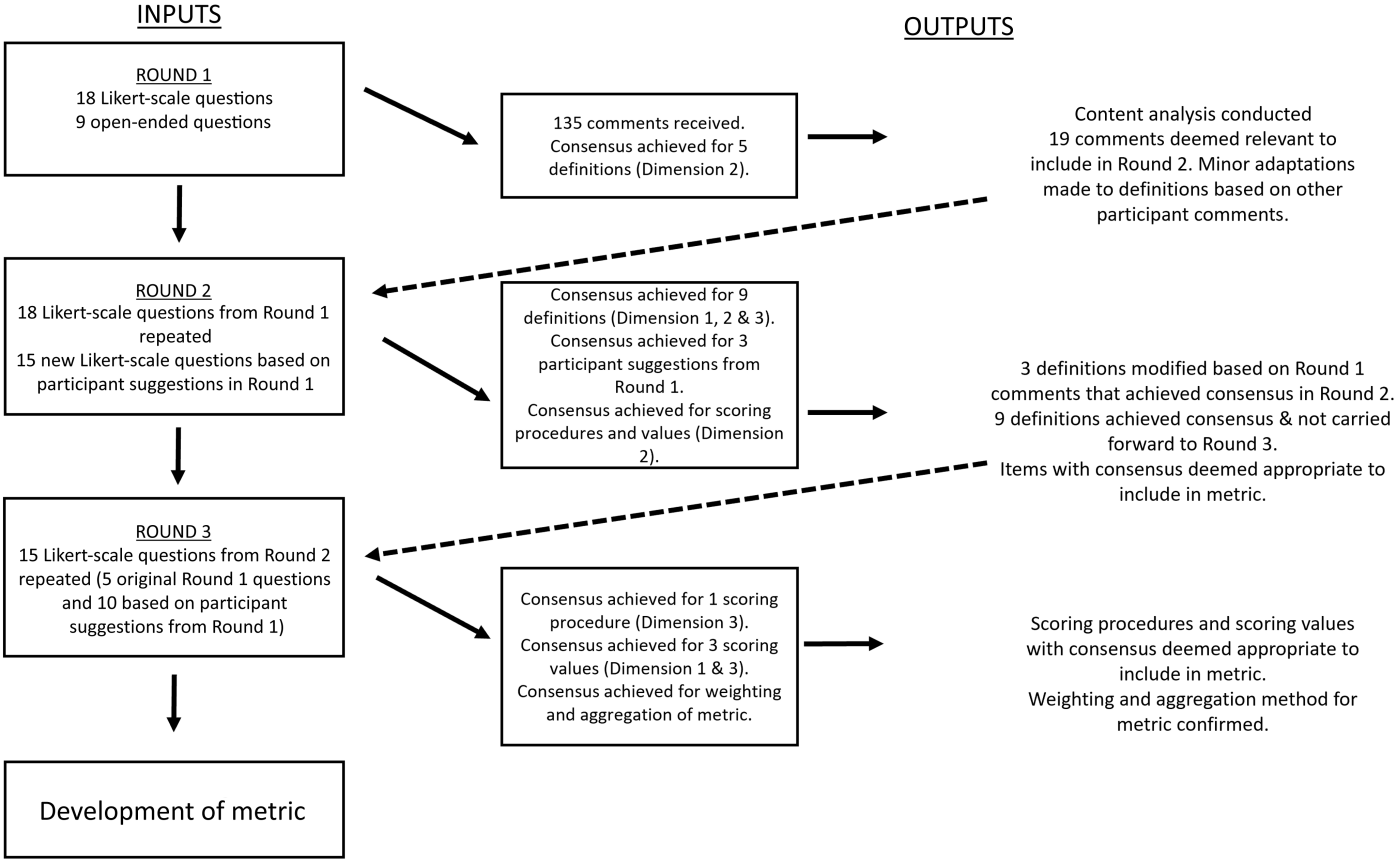
Figure 2 long description. Flow chart of Delphi method with inputs and outputs from each round adapted from Denniss et al.^(23)^.

In total, twenty-seven eligible experts consented to participate (35 % response rate from the seventy-eight experts invited to participate), of whom twenty-three (85 %) completed Round 1, fifteen (56 %) completed Round 2 and thirteen (48 %) completed Round 3. Most participants had expertise in food and nutrition policy and nutritional epidemiology. All participants who completed Round 3 had at least 5 years of experience, and 54 % had ≥15 years of experience (Table 2). The Delphi process is summarised in Figure 2.

**Table 2.** Participant characteristics across three Delphi surveys Table 2 long description.

Characteristics	Round 1 (*n* 23)		Round 2 (*n* 15)		Round 3 (*n* 13)	
	*n*	%	*n*	%	*n*	%
**Area of expertise** *						
Food and nutrition policy	17	74	12	80	11	85
Nutritional epidemiology	8	35	6	40	5	39
Dietary assessment	6	26	4	27	4	31
Environmental health	5	22	4	27	4	31
Environmental science and sustainability	1	4	0	0	0	0
Food pricing	1	4	0	0	0	0
Field*						
Academia/university	17	74	10	67	8	65
Non-government organisation	4	17	4	27	4	31
Government	3	13	2	13	2	16
**Years of experience**						
0–5	1	4	0	0	0	0
5–10	7	30	5	33	5	38
10–15	4	17	2	13	1	8
15–20	4	17	4	27	4	31
20+	7	30	4	27	3	23
**Highest level of education**						
Doctorate	20	87	12	80	10	77
Master’s degree	3	13	3	20	3	23
**Country**						
Australia	8	35	4	27	4	31
USA	5	22	3	20	3	23
UK	2	9	2	13	1	8
Canada	2	9	1	7	1	8
The Netherlands	2	9	2	13	2	15
Belgium	1	4	1	7	1	8
France	1	4	1	7	1	8
Israel	1	4	0	0	0	0
New Zealand	1	4	1	7	0	0

* Participants could fall under more than one category.

Participant characteristics differed between Round 1 and Round 3 due to attrition. Food and nutrition policy was the most reported area of expertise across all three surveys, and although six participants with this expertise dropped out, the proportion increased slightly (74 % in Round 1 and 85 % in Round 3). While there was dropout between Round 1 and Round 3 for participants with experience in nutritional epidemiology (*n* 3), dietary assessment (*n* 2) and environmental health (*n* 1), the proportion of participants from each group remained similar. One participant reported expertise in environmental science and food pricing, respectively, in Round 1; however, these participants did not complete Round 3. The number of participants with Master’s level education remained stable across all three surveys (*n* 3); however, half of the participants with a doctorate dropped out (*n* 10, 87 % in Round 1, 77 % in Round 3). Australia and the USA were the most represented geographical locations, and although approximately half of the Round 1 participants for each region dropped out, the proportion remained similar (Australia 35 % Round 1, 31 % Round 3 and USA 22 % Round 1, 23 % Round 3). There was one (4 %) participant from Israel and one (4 %) from New Zealand in Round 1, both of whom dropped out by Round 3, meaning these regions were not represented in the final survey.

### Round 1

In Round 1, participants rated eighteen Likert-scale questions, and participant agreement is summarised in Table 3. In total, 135 comments were received from the open-ended questions, of which nineteen were carried forward to Round 2 for participants to rate. Only definitions of eggs, poultry, fish, red meat and dietary variety achieved consensus. Some modification definitions were proposed in the open-ended questions, and these were carried into Round 2.

**Table 3.** Participant agreement^†^ about proposed diet quality score components across Delphi rounds Table 3 long description.

Proposed component of diet quality score	Round 1 % (Agree/strongly agree)	Round 2 % (Agree/strongly agree)	Round 3 % (Agree/strongly agree)
**Dimension 1: variety of unprocessed and minimally processed foods**			
*Definitions*			
Variety of plant foods	87 %	93 %	***
*Scoring procedure*			
Cut-off values	48 %	40 %	69 %
Comparison to population distribution*		33 %	31 %
*Scoring values*			
FAO MDD^(28)^	35 %	53 %	77 %
GDQS+^(34)^		53 %	93 %
**Dimension 2: intake of animal products**			
*Definitions*			
Egg	83 %	93 %**	***
Dairy products	65 %	80 %**	***
Poultry	83 %	93 %**	***
Fish	74 %	87 %**	***
Red meat	74 %	73 %**	***
*Scoring procedure*			
Cut-off values	57 %	60 %	***
Range of values (with upper and lower bounds)*		93 %	***
Comparison to population distribution*		33 %	***
*Scoring values*			
Egg ≤13 g/d	30 %	27 %	***
Dairy products ≤250 g/d	35 %	53 %	***
Poultry ≤29 g/d	39 %	47 %	***
Fish ≤28 g/d	39 %	40 %	***
Red meat ≤14 g/d	44 %	67 %	***
Scoring ranges*^(35)^		80 %	***
**Dimension 3: intake of UPF**			
*Definitions*			
Intake of UPF	52 %	87 %	***
*Scoring procedure*			
Cut-off values	43 %	60 %	62 %
Continuous score (e.g. 0–100 % of total intake)*		60 %	85 %
*Scoring values*			
≤10 % of daily dietary share	30 %	60 %	92 %
**Weighting and aggregation of the overall metric**			
Equal weighting of dimensions	48 %	47 %	85 %
UPF weighted more heavily*		7 %	0 %
Fruits and vegetables weighted more heavily*		40 %	31 %

FAO MDD, FAO Minimum Dietary Diversity; GDQS+, Global Diet Quality Score; UPF, ultra-processed foods.

† Consensus was set *a priori* at ≥70 % of participants selecting 1 or 2 on the scale (strongly disagree/disagree) or 4 or 5 (agree/strongly agree).

* Item suggested in open-ended response to Round 1 and added to Round 2 survey.

** Item adapted based on participant feedback in the preceding round.

*** Item not included in Round 3 because a consensus agreement achieved in the preceding round on item or contradictory item.

### Round 2

In Round 2, participants rated thirty-seven Likert-scale questions, of which nine achieved consensus (Table 3). In Round 1, participants provided feedback that some definitions should be modified. The following suggestions achieved consensus in Round 2: eggs should include liquid egg products (e.g. 100 % liquid eggs); pheasant should be included under the definition of poultry; fish should include seafood, with fish eggs included under this definition; and animal parts (e.g. pig trotters, chicken feet) should be included under the relevant definitions. Dairy products, red meat and poultry definitions presented in Round 1 achieved consensus in Round 2. Definitions that did not achieve consensus were included in Round 3 (Table 3).

In Round 1, participants suggested that there were not enough categories of foods to measure dietary diversity (based on the presented MDD measuring ten food groups^(28)^) and that the cut-off values were too low. There was also an overlap with animal products being included under MDD with recommended consumption not matching the recommendations from the EAT-Lancet planetary health diet, which was used to measure animal intake in Dimension 2. In response to this feedback, the Global Diet Quality Score healthy food groups (GDQS+)^(34)^ values for a variety of plant foods were included. GDQS+ was used to inform the variety dimension as it is the most recent metric designed to be sensitive to diet-related outcomes associated with both undernutrition and overnutrition globally. Despite consensus agreement regarding the definition for dietary variety (93 %), there was no consensus for using the GDQS+, and this suggestion was therefore included in Round 3. Participants suggested that processed foods (NOVA group 3, e.g. canned fruits and legumes) should be included in the definition of dietary variety. This suggestion achieved consensus (80 %). Participants provided feedback that it would be more appropriate to include a range for scoring small and moderate intakes of animal products, instead of one cut-off value. In response to this feedback, ranges for intakes of animal products as specified by Hanley-Cook et al.^(35)^ were included, and 80 % of participants agreed with this inclusion.

More than 85 % of participants agreed that the intake of UPF should be defined as the daily dietary share (energy or grams) of UPF. It was also proposed that restricting the intake of UPF should be defined as consuming ≤10 % of daily intake (grams or energy) from these foods. The cut-offs provided for participants to rate were based on the upper intakes of the reference group reported in a meta-analysis assessing the association between UPF consumption and all-cause mortality (intake of UPF in the reference group ranged from <4·7 % to <10 % of daily energy intake^(36)^). Based on feedback from Round 1, participants were also asked if the intake of UPF should be measured based on the dietary contribution (e.g. 0–100 % of total dietary intake). Neither achieved consensus, and both were repeated in Round 3. Participants suggested that the contribution of UPF subgroups to total UPF score should be encouraged to be reported.

In Round 1, there was no consensus that all three dimensions should be weighted equally when calculating an overall diet quality score. Some participants suggested that the intake of UPF should be weighted more heavily, and that intakes of fruits and vegetables should be weighted more than other foods within dietary variety. These questions did not reach consensus and were therefore included in Round 3.

### Round 3

In Round 3, participants rated fifteen Likert-scale questions, of which four achieved consensus (Table 3). For variety of plant foods, there was a greater level of agreement that the GDQS+ food groups and cut-offs should inform the diet quality score instead of the MDD (92 % *v.* 77 %). Therefore, the GDQS+ was chosen, and the MDD food groups and cut-off values were not used as indicators of dietary variety. For animal intake, there was no consensus that internal organs should not be included in the poultry food group; echinoderms (e.g. sea urchin) or whale and seal should be included under fish and seafood; pork should be captured under a separate category to red meat; and that game meat (e.g., kangaroo, wild boar and venison) should be captured under a different category to red meat. There was a greater level of agreement that a cut-off value for the intake of UPF should be used instead of a proportional measure (92 % *v.* 85 %), and therefore a cut-off was chosen as the most appropriate indicator for UPF intake.

There was no consensus that comparing intakes to population distribution is a better method than other approaches (e.g. specific upper and lower bounds from literature-based cut-offs) for scoring variety of plant foods, animal intake and UPF consumption. Finally, participants agreed that each should be equally weighted (85 %), disagreed that intake of UPF should be weighted more than the other dimensions (85 %), and there was no consensus that the intakes of fruits and vegetables should be weighted more than other foods within dietary variety.

### Indicators of sustainable healthy diets

There was agreement on eighteen food group indicators from the three dimensions of SHD (Table 4). Dimension 1 is comprised of twelve food groups, with food groups and scoring ranges informed by the GDQS+^(34)^. Dimension 2 is comprised of five food groups, with groups and scoring ranges informed by Hanley-Cook et al.^(35)^ and the EAT-Lancet planetary health diet^(29)^. Finally, Dimension 3 is comprised of one food group (UPF, NOVA Group 4^(26)^), and the scoring cut-off is based on a meta-analysis of the association between UPF and all-cause mortality^(36)^. There was consensus that each dimension should be equally weighted.

**Table 4.** Food group indicators of sustainable healthy diets and scoring procedures Table 4 long description.

Dimensions and food groups	Scoring procedures and values		
Dimension 1: Variety of unprocessed and minimally processed foods*	**Scoring range informed by GDQS+** ^(34)^ **(g/d)**		
Citrus fruits (e.g. orange, grapefruit)**	<24	24–69	>69
Deep orange fruits (e.g. apricot, papaya)**	<25	25–123	>123
Other fruits	<27	27–107	>107
Dark green leafy vegetables	<13	13–37	>37
Cruciferous vegetables	<13	13–36	>36
Deep orange vegetables	<9	9–45	>45
Other vegetables	<23	23–114	>114
Legumes (e.g. beans, lentils)	<9	9–42	>42
Deep orange tubers	<12	12–63	>63
Nuts and seeds	<7	7–13	>13
Whole grains	<8	8–13	>13
White roots and tubers	<27	27–107	>107
**Dimension 2: Animal products**	**Scoring range informed by Hanley-Cook et al.** ^(35)^ **(g/d)**		
Egg	<13	13–25	>25
Dairy products	<250	250–500	>500
Poultry	<29	29–58	>58
Fish and seafood	<28	28–100	>100
Red meat (beef, lamb and pork)	<14	14–28	>28
**Dimension 3: Ultra-processed foods**	**Cut-off value informed by Teneri et al.** ^(36)^ **(% energy/d)** ***		
Ultra-processed foods	≤10		

GDQS+, Global Diet Quality Score.

* Whole fruits and vegetables only does not include fruit or vegetable juices.

** Deep orange fruits that are citrus fruits are only captured under the citrus fruits group.

*** Continuous score (e.g. 0–100 % of total intake) also achieved consensus; however, a higher level of agreement was achieved for the use of the cut-off value, so the cut-off value was selected as the preferred scoring procedure.

## Discussion

This Delphi study aimed to achieve expert consensus regarding dietary indicators for the three key dimensions of sustainable diets: variety of unprocessed and minimally processed foods, intake of animal products and intake of UPF. The dimensions of SHD were agreed upon prior to this study^(4)^, and expert consensus was reached regarding the dietary indicators and scoring procedures for these dimensions. This study makes an important contribution to the field, because previously there was a lack of consensus on how to operationalise the dimensions of SHD. The agreed-upon dietary indicators, scoring and weighting procedures from this Delphi study have been used to inform the development of a multidimensional diet quality score, named SUSDIET, to measure the healthfulness and environmental sustainability of diets at the population level. The full SUSDIET metric, its comprehensive scoring procedures and its evaluation have been presented in a recent publication by our team^(37)^.

There was consensus that the three dimensions of SHD should be equally weighted in a diet quality score. In Round 3, 85 % of participants agreed that dietary variety, intake of animal products and UPF should be weighted equally, instead of weighting fruit and vegetable or UPF intakes more heavily. This finding is consistent with the FAO/WHO report on the guiding principles of SHD, which outlines these key dimensions as critical components of SHD but does not commit to any dimension as being of greater importance for sustainability or health^(4)^. Agreement regarding the equal contribution of the three dimensions is also consistent with the 3V-based diet to protect health and food system sustainability, which encompasses the same three dimensions^(16)^. Of the three rules of 3V-based diet (≤15 % energy from animal foods, ≤15 % energy from UPF and diverse dietary intake), none are given greater importance, and the authors state that exclusion of one of the dimensions threatens sustainability and human health^(16)^. This finding further underscores the limitations of extant metrics that do not adequately consider the three dimensions of SHD. Importantly, the equal weighting reflects consensus based on expert opinion and should be interpreted as a normative decision that the Delphi panel supported, rather than empirical evidence that each dimension has equal impact on health and environmental sustainability. Future studies should examine how these dimensions interact with one another, influence different outcomes and contribute to potential trade-offs.

Experts agreed that food group indicators of Dimension 1 (variety of unprocessed and minimally processed foods) were appropriate. The GDQS+^(34)^ food groups and intake value ranges were selected for scoring Dimension 1 and achieved a greater level of agreement as an appropriate scoring method compared with MDD cut-off values, although there was consensus agreement that the MDD values were also appropriate for scoring Dimension 1^(28)^. The GDQS+ has a greater number of plant-based food groups compared with the MDD (12 *v.* 7), with more specific groups in the GDQS+ (e.g. citrus fruits, cruciferous vegetables and deep orange vegetables), suggesting that it may be a more precise measure of diversity of plant food intake^(28,34)^. The GDQS+^(34)^ also provides more nuanced cut-offs based on levels of consumption, whereas the MDD^(28)^ is a simplified index that provides a binary assessment of consumption, regardless of the amount. Furthermore, the GDQS+ has been shown to perform well compared with the MDD in assessing nutrient adequacy and biochemical and anthropometric indicators of undernutrition using datasets from countries with varied income levels and cultures^(34)^, suggesting it is a robust measure to inform scoring of Dimension 1.

Similar to Dimension 1, participants agreed that Dimension 2 (intake of animal products) should be scored based on a range of values. The intake value ranges proposed by Hanley-Cook et al.^(35)^ were selected as the preferred scoring procedure for Dimension 2, over the EAT-Lancet cut-off values. The EAT-Lancet diet score has been critiqued due to the lack of minimum intake values for nutrient-dense food groups^(13,35)^. Hanley-Cook et al.^(35)^ proposed minimum intake values for the EAT-Lancet diet to prevent positively scoring non-consumption of food groups, which may be predictive of micronutrient inadequacy in low- and middle-income countries. Thus, the use of intake value ranges informed by Hanley-Cook et al.^(35)^ to score Dimension 2 are likely to be more appropriate. Furthermore, use of scoring ranges instead of cut-off values in a diet quality score may allow for more precise and sensitive assessment of Dimensions 1 and 2, whereby proportional scores can be easily applied.

There was consensus among the expert panel that Dimension 3 (intake of UPF) should be defined according to NOVA Group 4^(26)^ and measured with a cut-off value of ≤10 % dietary energy from UPF. A cut-off value to measure UPF intake is appropriate because UPF are not essential for health. To date, there is a lack of cut-off values for UPF intake proposed in the literature. National dietary guidelines that provide guidance about UPF intakes advise that UPF should be avoided or limited (e.g. Brazil, Malaysia) and do not provide an upper threshold of dietary energy^(38,39)^. To the knowledge of the authors, the only cut-off value proposed in the literature is ≤15 % energy in the 3V-based diet^(16)^. The threshold of 15 % is derived from six epidemiological studies (four cross-sectional) investigating UPF consumption and overweight/obesity risk^(16)^. The evidence base about UPF is rapidly growing, and the lower cut-off value of ≤10 % proposed in this study was derived from a more recent meta-analysis of forty prospective cohort studies assessing the association between UPF consumption and all-cause mortality^(36)^. The intake of UPF in the reference group ranged from <4·7 % to ≤10 % of total daily energy intake, and thus the value of ≤10 % was used^(36)^. This strategy follows a similar robust approach conducted to derive nutrient reference values^(40)^. While the value of ≤10 % was informed by recent evidence, alternative thresholds or graded scoring systems may also be valid and should be investigated in future research.

There are complex challenges for incorporating measurable indicators or proxies of all principles of SHD into a comprehensive dietary score. First, many of these indicators are relevant to food production and not food consumption. Second, there are challenges, convergences and divergences when simultaneously addressing health (e.g. undernutrition and diet-related non-communicable diseases), environmental sustainability and sociocultural aspects of diets. The burden and pragmatic aspects of operationalising all principles into a set of survey instruments are an obstacle, as the adoption of metrics is likely dependent on cultural appropriateness and resources available for monitoring and surveillance. Finally, dietary metrics are often developed using food groups as representations of constructs of diet quality, commonly measured using 24-h dietary recalls and FFQ, which poses a question on how to use food groups as proxies of the principles^(6)^. With this in mind, and in the absence of a gold standard to measure SHD, previous research has recommended that novel metrics could be developed considering food processing, dietary variety and intake of animal products as key dimensions for SHD when using food group consumption as representations of constructs of diet quality^(6)^. Considering this approach in dietary metric development has the potential to address at least eight principles of SHD (principles 1–7 and 9)^(6)^. These recommendations were considered when identifying and operationalising food group indicators in this study.

Findings from this study informed the development of SUSDIET, a multidimensional diet quality score for SHD^(37)^. SUSDIET was evaluated for construct and criterion validity using nationally representative dietary intake data from Australia, collected via 24-h recall. The metric was predictive of nutrient intakes and health outcomes, with SUSDIET scores being associated with a healthier nutrient profile, lower BMI, waist circumference, odds of obesity and abdominal obesity^(37)^.

This study has implications for advancing research. There is a need for a diet quality score to measure the sustainability and healthfulness of diets^(6)^. The dietary indicators and scoring procedures identified in this study have been used to inform the development of SUSDIET^(37)^. While SUSDIET predicted nutrient intakes and some health outcomes in our recent evaluation study, more research is required to validate the tool with environmental sustainability outcomes and a broader range of health outcomes. Although SUSDIET advances the assessment of healthy and sustainable diets by incorporating food processing, its comparative performance against other metrics (e.g. plant-based index, EAT-Lancet planetary health) to predict health and environmental sustainability outcomes requires further study. Further research will also be required to apply the score to different countries and food supply contexts and assess the feasibility of applying the scoring into different types of data (e.g. FFQ data, food records). SUSDIET was developed for the healthy free-living adult population, and ongoing research will be needed to adapt it for other populations such as the elderly, children, pregnant people and vegans.

This study also has implications for policy and practice. The Delphi findings and SUSDIET^(37)^ can inform the development of policies to promote SHD in regions and contexts that were well represented in this study (e.g., Australia, USA and other high-income countries). With adaptation depending on cultural contexts and surveillance data, the Delphi outcomes and SUSDIET can also support the evaluation of the impact of policies aimed at promoting SHD and for the monitoring and assessment of diets, which aligns with the WHO priority to build a global framework for monitoring healthy diets^(41)^. Finally, the dietary indicators that experts agreed upon can be used to inform dietary guidance and interventions aimed at mitigating threats to both human and environmental health. However, it is important to note that dietary metrics are not able to comprehensively capture all structural and systemic factors that influence sustainable diets (e.g., biodiversity, seasonality, equity, land use, food sovereignty, affordability), and this research operationalised sustainability through animal products, food processing and nutrient profiles. Therefore, the scope of how sustainability was operationalised should be considered when applying our findings in policy and practice.

This study has several strengths. To the knowledge of the authors, this is the first study to identify dietary indicators of dietary variety, intake of animal products and intake of UPF that can be used to inform the development of a diet quality score. This study is theoretically underpinned by the most recent high-level report published by FAO/WHO on SHD^(4)^, increasing its relevance. The Delphi findings should be interpreted as a refinement of these established evidence-based domains of SHD. The use of the Delphi method is another strength because it is a highly appropriate method for gaining consensus, and the sample size was suitable to produce meaningful results^(24)^. The majority of participants had ≥10 years of experience working in the field, were from multiple relevant disciplines (e.g. food and nutrition policy, nutritional epidemiology and environmental health) and had at least a Masters’ level education, indicating high level of knowledge of the content area.

This study has some limitations. Academics and respondents from Australia and the USA were over-represented, and there were no participants from Africa, Asia or South America, which may have limited the range of the perspectives captured. Participants were all from high-income countries, which may limit the generalisability of findings to low- and middle-income countries. There was attrition between surveys (56 % retention from Round 1). While attrition is common in Delphi studies^(21,30)^, it cannot be determined if attrition was random, and participants with diverging views may have been more inclined to drop out. Due to the introduction of new items and adaptation of extant items between survey rounds, the stability of consensus was not measured between each survey, which is consistent with the approach taken in previous Delphi studies^(23,33)^. However, this limits our ability to determine whether participant attrition introduced bias to the final consensus. The loss of a small number of participants with different expertise and regional contexts between surveys may have influenced the consensus outcomes because thirteen participants (a sufficient^(21)^, yet modest sample size) completed Round 3. The cut point for consensus of 70 % was informed by the literature and determined *a priori* as recommended^(21)^; however, it is important to note that selection of a different cut point may have yielded different outcomes. Although the dietary indicators were developed to reflect different cultural and food systems, scenario adaptations may be required in certain contexts. Dietary indicators may also require updates as the food supply continues to evolve, and novel forms of foods become more available. This study was underpinned by the FAO/WHO global report on SHD^(4)^, and the dietary indicators that were included in the Delphi surveys were evidence-based, which may help mitigate the impact of some of these limitations. Finally, in Delphi studies, consensus does not imply empirical correctness, and instead the results are indicative of expert opinion and what participants agreed upon; therefore, the indicators identified in this study and operationalised in SUSDIET^(37)^ should be tested, adapted and refined when applied in diverse populations and food systems.

### Conclusion

Consensus from experts led to the identification of indicators of an SHD to inform the development of a diet quality score intended for global application. The indicators incorporate a variety of unprocessed and minimally processed foods, intake of animal products and dietary contribution of UPF. There was consensus that food groups and scoring procedures for dietary variety should be informed by the GDQS+^(34)^; intake of animal products should be informed by Hanley-Cook et al.^(35)^ and the EAT-Lancet planetary health diet^(29)^; UPF intake should be informed by the NOVA system (Group 4)^(26)^ and the meta-analysis by Taneri et al.^(36)^; and all three dimensions should be equally weighted in a diet quality score. Findings from this study will be used to inform the development of a multidimensional diet quality score and enable research aiming to assess the impact of diets on both health and environmental sustainability outcomes among the general adult population.

## Supporting information

Denniss et al. supplementary material Denniss et al. supplementary material

## Supplementary material

To view supplementary material for this article, please visit https://doi.org/10.1017/S0007114526106345

## Figure 2 Long description

The flowchart illustrates the Delphi method used to develop a metric for sustainable healthy diets, detailing inputs, outputs, and consensus achieved in each round. Panel A: Round 1 involves 18 Likert-scale questions and 9 open-ended questions. Outputs include 135 comments received, with consensus achieved for 5 definitions in Dimension 2. Content analysis is conducted, with 19 comments deemed relevant for Round 2 and minor adaptations made to definitions based on other participant comments. Panel B: Round 2 involves repeating 18 Likert-scale questions from Round 1 and adding 15 new Likert-scale questions based on participant suggestions. Outputs include consensus achieved for 9 definitions in Dimensions 1, 2, and 3, consensus for 3 participant suggestions from Round 1, and consensus for scoring procedures and values in Dimension 2. Three definitions are modified based on Round 1 comments that achieved consensus in Round 2, and 9 definitions achieved consensus and are carried forward to Round 3. Items with consensus are deemed appropriate to include in the metric. Panel C: Round 3 involves repeating 15 Likert-scale questions from Round 2, with 5 original questions and 10 based on participant suggestions from Round 1. Outputs include consensus achieved for 1 scoring procedure in Dimension 3, consensus for 3 scoring values in Dimensions 1 and 3, and consensus for weighting and aggregation of the metric. Scoring procedures and values with consensus are deemed appropriate to include in the metric, and the weighting and aggregation method for the metric is confirmed.

Navigate back to Figure 2.

## Table 1 Long description

A table defining and describing dimensions of sustainable healthy diets. The table has two main sections: Definitions and Dimensions of sustainable healthy diets. The Definitions section includes four rows and two columns. Column headers are Definitions and Description. Row 1: Sustainable healthy diets, Dietary patterns that promote all dimensions of health and wellbeing; have low environmental pressure and impact; are accessible, affordable, safe and equitable; and are culturally acceptable. Row 2: Dietary patterns, Consider the combinations, types and amounts of foods consumed in the diet on a regular basis. Row 3: Dietary metric/diet quality score, Measures of investigator-defined food-based dietary patterns (also referred to as a priori methods). Reflect previous knowledge, guidelines and recommendations using pre-specified criteria, for example, adherence to health and/or environmental sustainability-based recommendations. Row 4: Unprocessed and minimally processed foods, Defined within the NOVA system as edible parts of plants, animals, fungi, algae and water, and natural foods altered by processes designed to preserve natural foods, to make them suitable for storage, or to make them safe, edible or more pleasant to consume without adding food substances to the original food. The Dimensions of sustainable healthy diets section includes three rows and two columns. Column headers are Dimension and Description. Row 1: Dimension 1, Sustainable healthy diets are based on a great variety of unprocessed or minimally processed foods. Row 2: Dimension 2, Sustainable healthy diets can include moderate amounts of eggs, dairy products, poultry and fish and small amounts of red meat. Row 3: Dimension 3, Sustainable healthy diets restrict consumption of ultra-processed foods.

Navigate back to Table 1.

## Table 2 Long description

The table presents participant characteristics across three Delphi surveys, detailing area of expertise, field, years of experience, highest level of education, and country. It has 23 rows and 11 columns. The columns are labeled Round 1 (n 23), Round 2 (n 15), and Round 3 (n 13), with sub-columns for count (n) and percentage (%). The rows are grouped under characteristics such as area of expertise, field, years of experience, highest level of education, and country. Each row lists the specific categories within these characteristics. For example, under area of expertise, categories include food and nutrition policy, nutritional epidemiology, dietary assessment, environmental health, environmental science and sustainability, and food pricing. The table shows the count and percentage of participants in each category for the three rounds. Notable trends include a high percentage of participants with expertise in food and nutrition policy and a significant number of participants with a doctorate degree. The table also indicates the distribution of participants across different fields, years of experience, and countries.

Navigate back to Table 2.

## Table 3 Long description

A table comparing participant agreement on diet quality score components across three rounds. The table has three main dimensions: variety of unprocessed and minimally processed foods, intake of animal products, and intake of ultra-processed foods (UPF). Each dimension includes definitions, scoring procedures, and scoring values. The table has 25 rows and 4 columns. Column headers are Round 1 percent (Agree/strongly agree), Round 2 percent (Agree/strongly agree), and Round 3 percent (Agree/strongly agree). Row labels include various components such as variety of plant foods, cut-off values, comparison to population distribution, and specific food items like egg, dairy products, poultry, fish, and red meat. Notable trends include increased agreement in Round 2 and Round 3 compared to Round 1 for most components. For example, the variety of plant foods agreement increased from 87 percent in Round 1 to 93 percent in Round 2. The intake of UPF showed a significant increase in agreement from 52 percent in Round 1 to 87 percent in Round 2. The table also includes specific scoring values and ranges for different food items and UPF intake.

Navigate back to Table 3.

## Table 4 Long description

A table with three dimensions and food groups, scoring procedures and values. Dimension 1: Variety of unprocessed and minimally processed foods. Scoring range informed by GDQS+ (g/d). Row 1: Citrus fruits, <24, 24-69, >69. Row 2: Deep orange fruits, <25, 25-123, >123. Row 3: Other fruits, <27, 27-107, >107. Row 4: Dark green leafy vegetables, <13, 13-37, >37. Row 5: Cruciferous vegetables, <13, 13-36, >36. Row 6: Deep orange vegetables, <9, 9-45, >45. Row 7: Other vegetables, <23, 23-114, >114. Row 8: Legumes, <9, 9-42, >42. Row 9: Deep orange tubers, <12, 12-63, >63. Row 10: Nuts and seeds, <7, 7-13, >13. Row 11: Whole grains, <8, 8-13, >13. Row 12: White roots and tubers, <27, 27-107, >107. Dimension 2: Animal products. Scoring range informed by Hanley-Cook et al. (g/d). Row 1: Egg, <13, 13-25, >25. Row 2: Dairy products, <250, 250-500, >500. Row 3: Poultry, <29, 29-58, >58. Row 4: Fish and seafood, <28, 28-100, >100. Row 5: Red meat, <14, 14-28, >28. Dimension 3: Ultra-processed foods. Cut-off value informed by Teneri et al. (% energy/d). Row 1: Ultra-processed foods, <=10.

Navigate back to Table 4.
